# Supplementary material for: Viruses Ubiquity and Diversity in Atacama Desert Endolithic Communities
Source: Viruses. 2022 Sep 7;14(9):1983. doi: 10.3390/v14091983 (PMC9500819; doi:10.3390/v14091983)
Supplement: Supplementary file 1 [file viruses-14-01983-s001.zip › viruses-1888714-supplementary.pdf]

SUPPLEMENTARY MATERIAL

Viruses Ubiquity and Diversity in Atacama Desert Endolithic Communities

Leora Busse, Mike Tisza, Jocelyne DiRuggiero

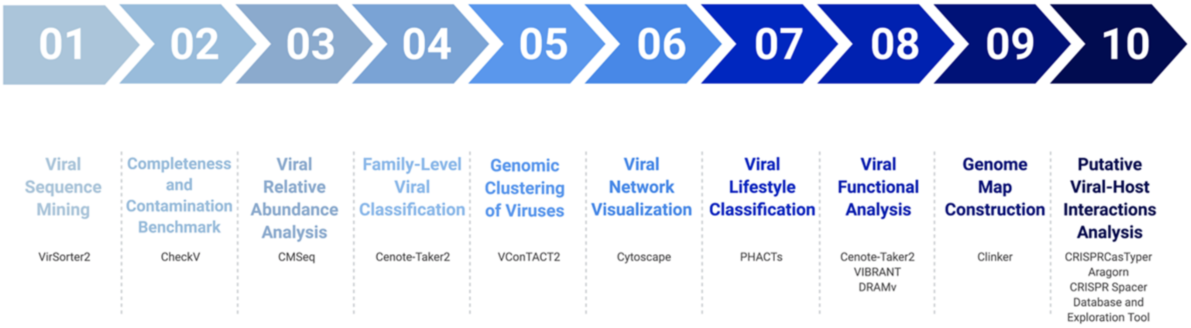

Figure S1. Computational pipeline and tools used to analyze the viromes in this study.

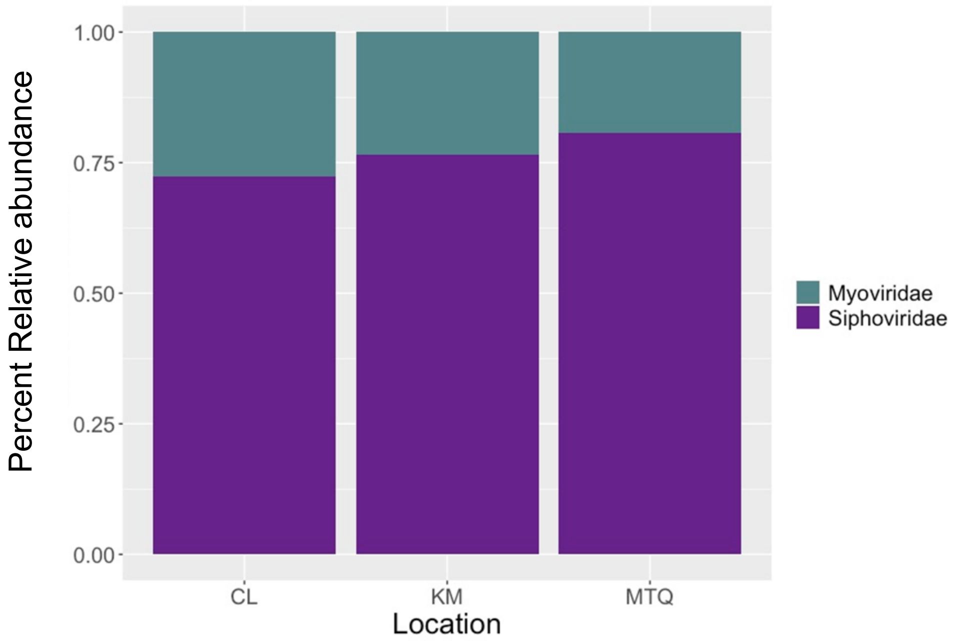

Figure S2. Relative abundances of viral families identified in the gypsum metagenomes from samples collected from the CL, KM, and MTQ sites.

A

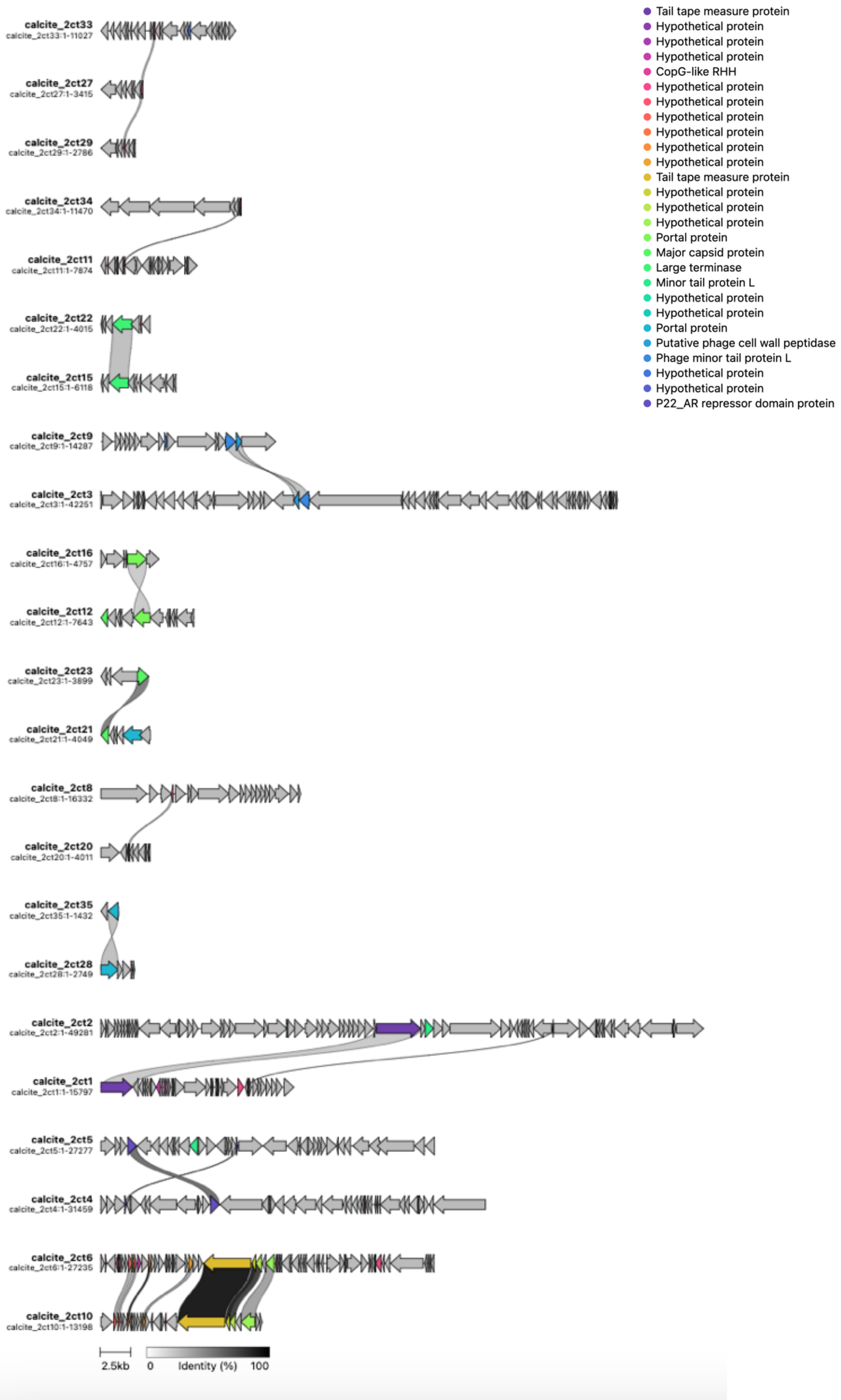

calcite_2ct33
calcite_2ct33:1-11027
calcite_2ct27
calcite_2ct27:1-3415
calcite_2ct29
calcite_2ct29:1-2786
calcite_2ct34
calcite_2ct34:1-11470
calcite_2ct11
calcite_2ct11:1-7874
calcite_2ct22
calcite_2ct22:1-4015
calcite_2ct15
calcite_2ct15:1-6118
calcite_2ct9
calcite_2ct9:1-14287
calcite_2ct3
calcite_2ct3:1-42251
calcite_2ct16
calcite_2ct16:1-4757
calcite_2ct12
calcite_2ct12:1-7643
calcite_2ct23
calcite_2ct23:1-3899
calcite_2ct21
calcite_2ct21:1-4049
calcite_2ct8
calcite_2ct8:1-16332
calcite_2ct20
calcite_2ct20:1-4011
calcite_2ct35
calcite_2ct35:1-1432
calcite_2ct28
calcite_2ct28:1-2749
calcite_2ct2
calcite_2ct2:1-49281
calcite_2ct1
calcite_2ct1:1-15797
calcite_2ct5
calcite_2ct5:1-27277
calcite_2ct4
calcite_2ct4:1-31459
calcite_2ct6
calcite_2ct6:1-27235
calcite_2ct10
calcite_2ct10:1-13198
2.5kb
0
Identity (%)
100
Tail tape measure protein
Hypothetical protein
Hypothetical protein
Hypothetical protein
CopG-like RHH
Hypothetical protein
Hypothetical protein
Hypothetical protein
Hypothetical protein
Hypothetical protein
Hypothetical protein
Tail tape measure protein
Hypothetical protein
Hypothetical protein
Hypothetical protein
Portal protein
Major capsid protein
Large terminase
Minor tail protein L
Hypothetical protein
Hypothetical protein
Portal protein
Putative phage cell wall peptidase
Phage minor tail protein L
Hypothetical protein
Hypothetical protein
P22_AR repressor domain protein

B

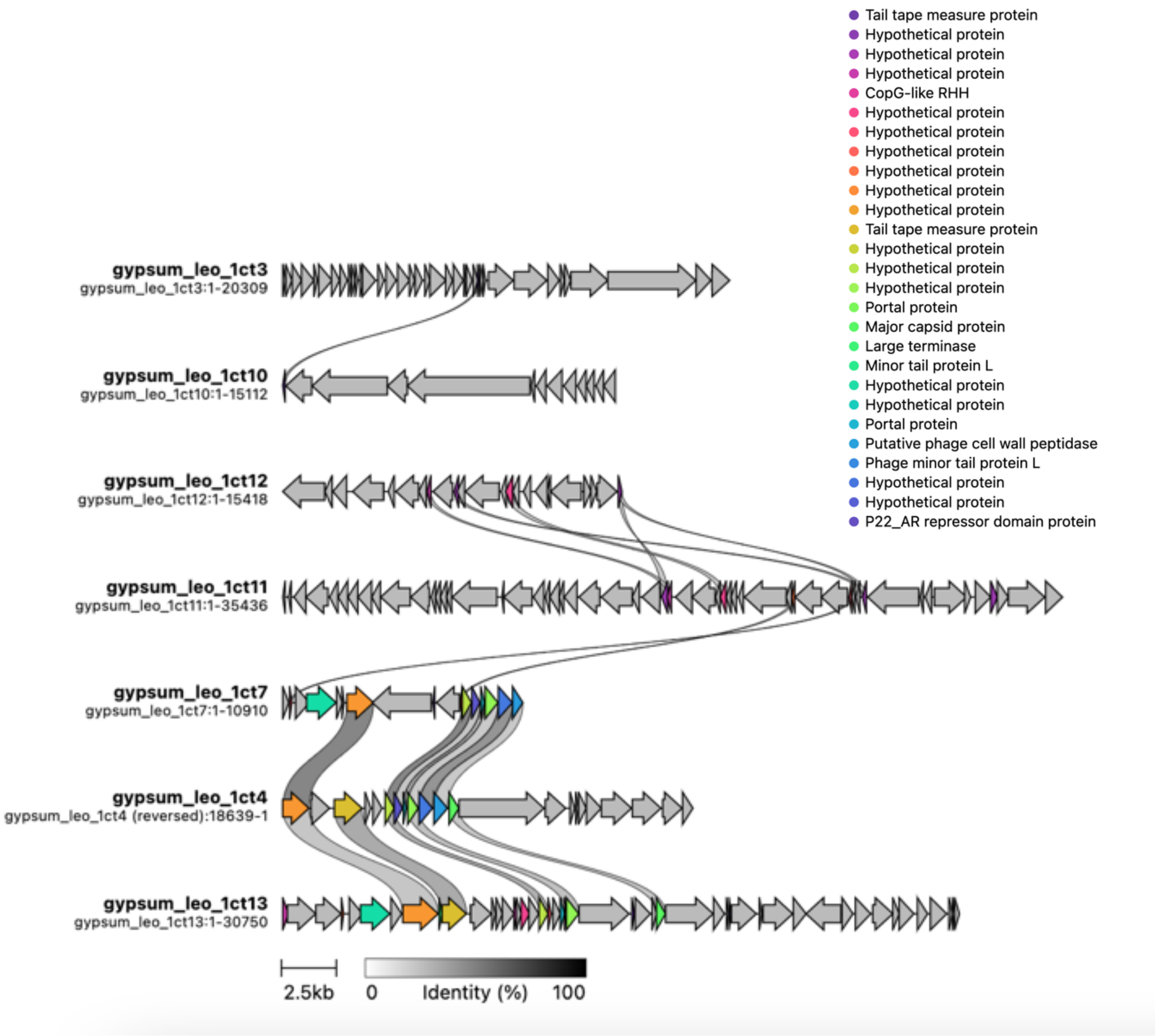
Tail tape measure protein
Hypothetical protein
Hypothetical protein
Hypothetical protein
CopG-like RHH
Hypothetical protein
Hypothetical protein
Hypothetical protein
Hypothetical protein
Hypothetical protein
Hypothetical protein
Tail tape measure protein
Hypothetical protein
Hypothetical protein
Hypothetical protein
Portal protein
Major capsid protein
Large terminase
Minor tail protein L
Hypothetical protein
Hypothetical protein
Portal protein
Putative phage cell wall peptidase
Phage minor tail protein L
Hypothetical protein
Hypothetical protein
P22_AR repressor domain protein
gypsum_leo_1ct3
gypsum_leo_1ct3:1-20309
gypsum_leo_1ct10
gypsum_leo_1ct10:1-15112
gypsum_leo_1ct12
gypsum_leo_1ct12:1-15418
gypsum_leo_1ct11
gypsum_leo_1ct11:1-35436
gypsum_leo_1ct7
gypsum_leo_1ct7:1-10910
gypsum_leo_1ct4
gypsum_leo_1ct4 (reversed):18639-1
gypsum_leo_1ct13
gypsum_leo_1ct13:1-30750
2.5kb
0
Identity (%)
100

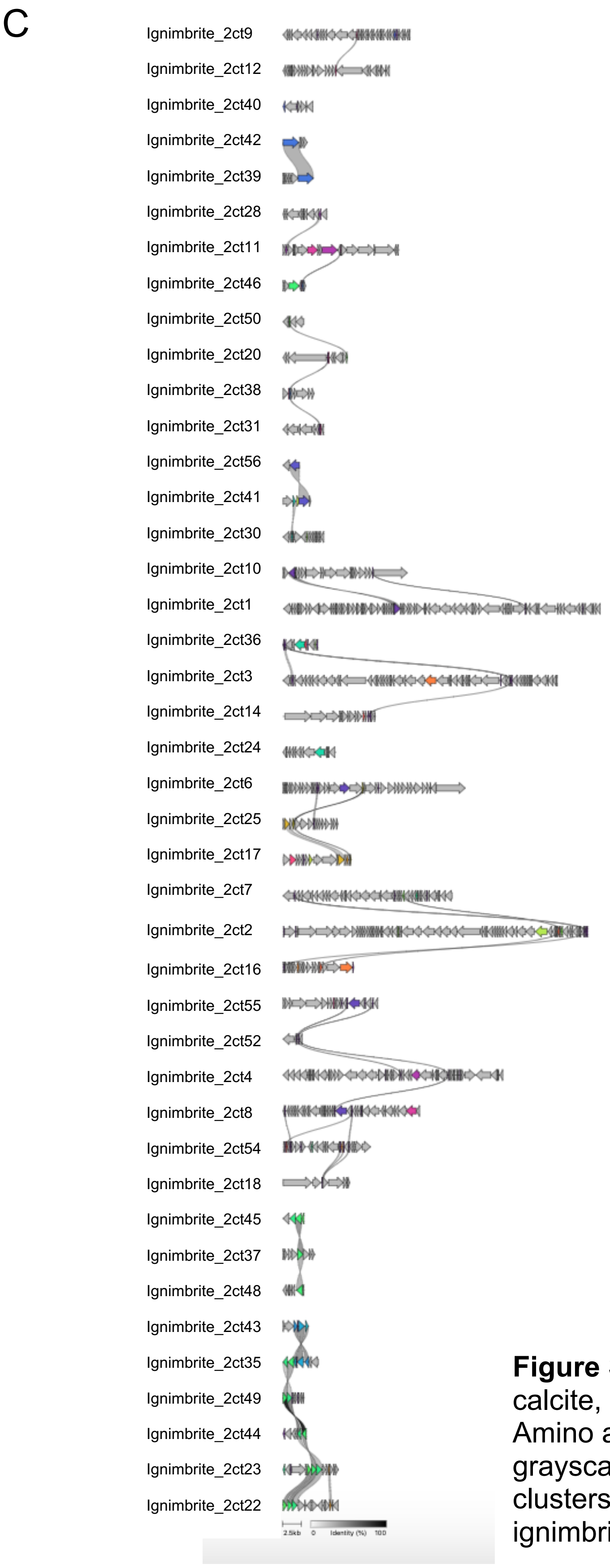

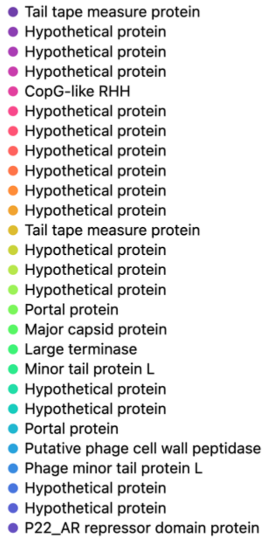

Figure S3. Genome maps of all viruses in calcite, gypsum, and ignimbrite substrates. Amino acids identity is indicated by the grayscale. Maps were drawn with Clinker. Viral clusters found in (A) calcite, (B) gypsum, and (C) ignimbrite (zoom in to see details).

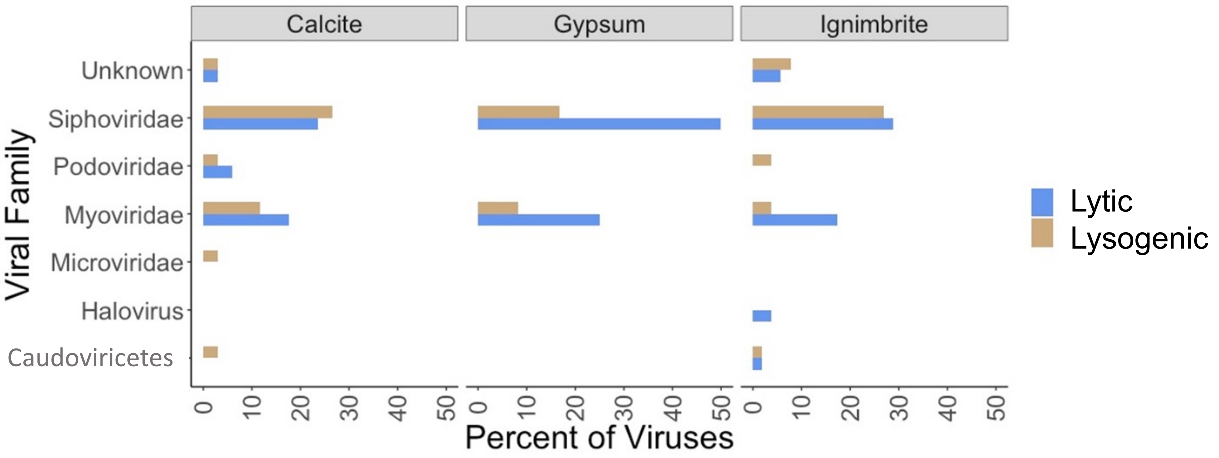

Figure S4. Relative abundances of viral families with lytic or lysogenic life cycles in the calcite, gypsum, and ignimbrite metagenomes.

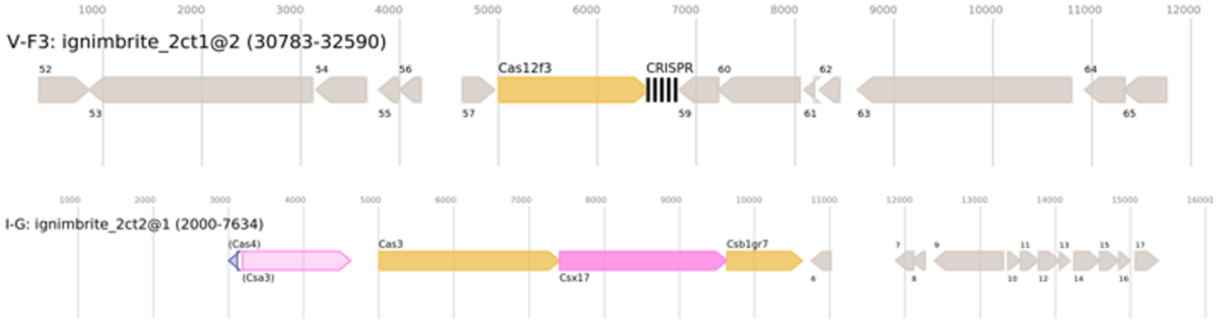

Figure S5. Viral genome maps showing CRISPR domains identified with CRISPRCasTyper for two viruses from the ignimbrite metagenomes. The striped black and white box shows a CRISPR array with numbers of spacer/repeat sequences. In yellow are CAS genes, in purple are accessory genes, and in light blue and light pink are genes with alignment scores below the thresholds. Unknown genes are in gray.

Table S1. Metagenomic libraries description

Substrate	Location	Number of Libraries	Assembled Libraries Total Size (Gbp)	Number of Contigs in Assembled Libraries	Accession Number[1]
Calcite	Valle de la Luna (VL)	3	217	81,607	3300039108
Gypsum	Cordon de Lila (CL)	3	320	120,629	3300028913
	KM37 (KM)	3			
	Monturaqui (MTQ)	3			
Ignimbrite	Monturaqui (MTQ)	3	164	50,371	3300039169

[1]JGI Taxon OID number

Table S2. Viral genomes identified in the three endolithic substrates

	VirSorter2			CheckV			Cenote-Taker2		CMSeq
Substrate	Contig ID	Coverage	Length (kbp)	Quality	Completeness Percentage	Contamination	ID	Number of Hallmark Genes	Abundance
Gypsum	k141_144888	Full	24.963	Low	21.65	0	gypsum_leo_1ct2	7	17
Gypsum	k141_658652	Full	20.309	Low	26.15	0	gypsum_leo_1ct3	2	19
Gypsum	k141_297749	Full	18.639	Low	21.95	36.03	gypsum_leo_1ct4	7	21
Gypsum	k141_656469	Full	18.435	Low	34.49	0	gypsum_leo_1ct5	5	24
Gypsum	k141_520133	Full	13.922	Low	37.26	0	gypsum_leo_1ct6	7	24
Gypsum	k141_103458	Full	10.91	Low	25.93	0	gypsum_leo_1ct7	4	50
Gypsum	k141_387731	Full	9.69	Low	26.93	0	gypsum_leo_1ct8	4	48
Gypsum	k141_138735	Full	9.361	Low	26.36	0	gypsum_leo_1ct9	3	13
Gypsum	k141_505949	Full	15.112	Low	20.58	0	gypsum_leo_1ct10	5	18
Gypsum	k141_657276	Partial	35.436	Low	41.62	35.26	gypsum_leo_1ct11	7	84
Gypsum	k141_373844	Partial	15.418	Medium	73	26.92	gypsum_leo_1ct12	3	380
Gypsum	k141_642343	Partial	30.75	Low	21.96	45.01	gypsum_leo_1ct13	7	15
Calcite	Ga0397528_00778	Full	15.797	Low	17.05	0	calcite_2ct1	1	12
Calcite	Ga0397528_00045	Full	49.281	High	98.79	0	calcite_2ct2	8	16
Calcite	Ga0397528_00071	Full	42.251	High	100	0	calcite_2ct3	9	11
Calcite	Ga0397528_00160	Full	31.459	Low	31.28	0	calcite_2ct4	5	21

Calcite	Ga0397528_00210	Full	27.277	Low	30.13	0	calcite_2ct5	4	12
Calcite	Ga0397528_00222	Full	27.235	Low	27.88	0	calcite_2ct6	1	24
Calcite	Ga0397528_00468	Full	19.239	Low	25.44	0	calcite_2ct7	1	8
Calcite	Ga0397528_00664	Full	16.332	Low	24.6	0	calcite_2ct8	2	12
Calcite	Ga0397528_00962	Full	14.287	Low	17.89	0	calcite_2ct9	4	12
Calcite	Ga0397528_01098	Full	13.198	Low	19.22	0	calcite_2ct10	1	9
Calcite	Ga0397528_03303	Full	7.874	Low	7.22	0	calcite_2ct11	1	13
Calcite	Ga0397528_03553	Full	7.643	Low	13.16	0	calcite_2ct12	3	11
Calcite	Ga0397528_04289	Full	6.948	Low	16.23	0	calcite_2ct13	0	9
Calcite	Ga0397528_05171	Full	6.324	Low	9.35	0	calcite_2ct14	4	9
Calcite	Ga0397528_05222	Full	6.118	Low	14.93	0	calcite_2ct15	1	10
Calcite	Ga0397528_08206	Full	4.757	Low	8.39	0	calcite_2ct16	2	33
Calcite	Ga0397528_08263	Full	4.414	Low	5.52	0	calcite_2ct17	1	9
Calcite	Ga0397528_08502	Full	4.29	Low	5.9	0	calcite_2ct18	0	11
Calcite	Ga0397528_10284	Full	4.308	Low	6.48	0	calcite_2ct19	4	485
Calcite	Ga0397528_10994	Full	4.011	Low	5.29	0	calcite_2ct20	0	24
Calcite	Ga0397528_11374	Full	4.049	Low	26.43	0	calcite_2ct21	2	12
Calcite	Ga0397528_11519	Full	4.015	Low	8.09	0	calcite_2ct22	1	33
Calcite	Ga0397528_11774	Full	3.899	Low	25.44	0	calcite_2ct23	1	15
Calcite	Ga0397528_12887	Full	2.745	Low	7.77	0	calcite_2ct24	1	13
Calcite	Ga0397528_13622	Full	3.609	Low	6.48	0	calcite_2ct25	1	12
Calcite	Ga0397528_13811	Full	3.58	Low	8.55	0	calcite_2ct26	2	20
Calcite	Ga0397528_14864	Full	3.415	Low	6.87	0	calcite_2ct27	3	26
Calcite	Ga0397528_20264	Full	2.749	Low	6.74	0	calcite_2ct28	1	28
Calcite	Ga0397528_20302	Full	2.786	Low	9.39	0	calcite_2ct29	1	14
Calcite	Ga0397528_22128	Full	2.59	Low	5.36	0	calcite_2ct30	3	5
Calcite	Ga0397528_06764	Partial	1.799	Low	29.71	0	calcite_2ct31	1	5
Calcite	Ga0397528_00540	Partial	12.712	Low	8.79	0	calcite_2ct32	1	7
Calcite	Ga0397528_00674	Partial	11.027	Low	12.68	0	calcite_2ct33	2	17
Calcite	Ga0397528_01028	Partial	11.47	Low	5.97	0	calcite_2ct34	2	17
Calcite	Ga0397528_51335	Lt2gene	1.432	Low	9.37	0	calcite_2ct35	1	23
Ignimbrite	Ga0397530_00081	Full	42.345	Medium	68.53	0	ignimbrite_2ct1	6	30
Ignimbrite	Ga0397530_00089	Full	40.679	Medium	64.04	0	ignimbrite_2ct2	7	38
Ignimbrite	Ga0397530_00124	Full	36.584	Medium	70.77	0	ignimbrite_2ct3	9	9
Ignimbrite	Ga0397530_00351	Full	24.218	Low	36.16	0	ignimbrite_2ct5	5	36
Ignimbrite	Ga0397530_00420	Full	24.318	Low	29.95	0	ignimbrite_2ct6	5	20
Ignimbrite	Ga0397530_00495	Full	22.567	Medium	54.12	0	ignimbrite_2ct7	4	15
Ignimbrite	Ga0397530_00759	Full	18.232	Low	27.48	0	ignimbrite_2ct8	2	38
Ignimbrite	Ga0397530_00918	Full	16.927	Low	8.46	0	ignimbrite_2ct9	0	91

Ignimbrite	Ga0397530_00940	Full	16.584	Low	44.23	0	ignimbrite_2ct10	6	90
Ignimbrite	Ga0397530_01030	Full	15.516	Low	23.16	0	ignimbrite_2ct11	4	28
Ignimbrite	Ga0397530_01189	Full	14.184	Low	8.04	0	ignimbrite_2ct12	1	257
Ignimbrite	Ga0397530_01215	Full	14.798	Low	19.1	0	ignimbrite_2ct13	6	13
Ignimbrite	Ga0397530_01682	Full	12.382	Low	16.34	0	ignimbrite_2ct14	0	14
Ignimbrite	Ga0397530_01739	Full	12.084	Low	23.82	0	ignimbrite_2ct15	0	13
Ignimbrite	Ga0397530_02667	Full	9.447	Low	20.11	0	ignimbrite_2ct16	2	10
Ignimbrite	Ga0397530_02884	Full	9.073	Low	12.89	0	ignimbrite_2ct17	1	38
Ignimbrite	Ga0397530_02943	Full	8.904	Low	10.28	0	ignimbrite_2ct18	0	12
Ignimbrite	Ga0397530_03159	Full	8.588	Low	10.34	0	ignimbrite_2ct20	1	8
Ignimbrite	Ga0397530_03251	Full	8.432	Low	35.53	0	ignimbrite_2ct21	2	8
Ignimbrite	Ga0397530_03802	Full	7.35	Low	6.54	0	ignimbrite_2ct22	1	19
Ignimbrite	Ga0397530_04089	Full	7.389	Low	6.57	0	ignimbrite_2ct23	1	11
Ignimbrite	Ga0397530_04141	Full	7.332	Low	9.55	0	ignimbrite_2ct24	1	7
Ignimbrite	Ga0397530_04153	Full	7.319	Low	10.02	0	ignimbrite_2ct25	1	21
Ignimbrite	Ga0397530_04315	Full	7.128	Low	12.57	0	ignimbrite_2ct26	1	6
Ignimbrite	Ga0397530_05470	Full	5.847	Low	7.71	0	ignimbrite_2ct28	2	9
Ignimbrite	Ga0397530_05700	Full	5.974	Low	9.08	0	ignimbrite_2ct29	3	8
Ignimbrite	Ga0397530_06523	Full	5.422	Low	12.83	0	ignimbrite_2ct30	1	13
Ignimbrite	Ga0397530_06571	Full	5.395	Low	5.96	0	ignimbrite_2ct31	3	11
Ignimbrite	Ga0397530_06661	Full	5.306	Low	8.23	0	ignimbrite_2ct32	1	27
Ignimbrite	Ga0397530_06738	Full	5.231	Low	12.45	0	ignimbrite_2ct33	4	8
Ignimbrite	Ga0397530_07734	Full	4.789	Low	7.67	0	ignimbrite_2ct34	2	12
Ignimbrite	Ga0397530_07990	Full	4.694	Low	7.73	0	ignimbrite_2ct35	3	8
Ignimbrite	Ga0397530_08209	Full	4.618	Low	8.82	0	ignimbrite_2ct36	2	28
Ignimbrite	Ga0397530_08487	Full	4.3	Low	9.87	0	ignimbrite_2ct37	1	12
Ignimbrite	Ga0397530_09499	Full	4.146	Low	6.2	0	ignimbrite_2ct38	1	8
Ignimbrite	Ga0397530_09804	Full	4.049	Low	8.62	0	ignimbrite_2ct39	1	11
Ignimbrite	Ga0397530_10083	Full	3.969	Low	5.94	0	ignimbrite_2ct40	1	6
Ignimbrite	Ga0397530_10466	Full	3.712	Low	8.57	0	ignimbrite_2ct41	1	7
Ignimbrite	Ga0397530_10580	Full	3.238	Low	6.74	0	ignimbrite_2ct42	1	21
Ignimbrite	Ga0397530_12410	Full	3.374	Low	6.05	0	ignimbrite_2ct43	2	6
Ignimbrite	Ga0397530_14057	Full	3.081	Low	8.71	0	ignimbrite_2ct44	1	33
Ignimbrite	Ga0397530_14239	Full	2.775	Low	6.24	0	ignimbrite_2ct45	1	7
Ignimbrite	Ga0397530_14393	Full	3.022	Low	7.26	0	ignimbrite_2ct46	1	5
Ignimbrite	Ga0397530_14629	Full	2.978	Low	6.34	0	ignimbrite_2ct47	1	8
Ignimbrite	Ga0397530_15383	Full	2.859	Low	10.01	0	ignimbrite_2ct48	1	9
Ignimbrite	Ga0397530_15895	Full	2.785	Low	6.9	0	ignimbrite_2ct49	1	7
Ignimbrite	Ga0397530_15988	Full	2.771	Low	5.15	0	ignimbrite_2ct50	2	5

Ignimbrite	Ga0397530_16319	Full	2.72	Low	5.6	0	ignimbrite_2ct51	1	5
Ignimbrite	Ga0397530_17695	Full	2.548	Low	6.08	0	ignimbrite_2ct52	0	25
Ignimbrite	Ga0397530_18692	Full	2.432	Low	8.29	0	ignimbrite_2ct53	1	27
Ignimbrite	Ga0397530_00096	Partial	11.677	Low	19.65	0	ignimbrite_2ct54	1	27
Ignimbrite	Ga0397530_00308	Partial	12.629	Low	25.76	0	ignimbrite_2ct55	1	11
Ignimbrite	Ga0397530_21188	lt2gene	2.188	Low	5.01	0	ignimbrite_2ct56	1	4
	Total								2911

Table S3. Metabolic categories for viral auxiliary metabolic genes (AMGs) identified by DRAMv

Substrate	Number of AMGs	Identifiable AMG Categories
Calcite	7	• Pyrimidine deoxyribonucleotide biosynthesis
Gypsum	5	• Glucuronate pathway<br>• Nucleotide sugar biosynthesis<br>• Pyrimidine deoxyribonucleotide biosynthesis
Ignimbrite	1	Organic Nitrogen<br>• Methionine degradation

File S1. Analysis pipelines and visualization scripts used in this study

```
#Endolithic viruses scripts and links to github or bitbucket pages for
programs

#VirSorter2
#link: https://github.com/jiarong/VirSorter2
virsorter run -w viruses.out -i ignimbrite_coassembled_contigs.fna —include-
groups “dsDNAphage,NCLDV,ssDNA, lavidaviridae” -j 4 —min-score 0.75 —
hallmark-required —hallmark-required-on-short —min-length 3
#repeat for each rock type

#CheckV
#link: https://bitbucket.org/berkeleylab/checkv/src/master/
#to keep the sequences that are >5% complete, used the following commands
checkv end_to_end calcite_aa.fasta output_directory -t 16 -d checkv-db-v0.6
bioawk -c fastx ‘{print “>”$name ; print $seq}’ my_contigs.fasta >
my_contigs.format.fasta
tail -n+2 quality_summary.tsv | awk ‘{ if ($10>5 && $10!=“NA”) {print $1}}’ >
my_study_contig_names_over5checkv.txt
grep -A1 -Ff my_study_contig_names_over5checkv.txt
my_contigs.format.fasta | sed ‘/- -/d’ > my_contigs_over5checkv.fasta
#repeat for each rock type

#vConTACT2
#link: https://bitbucket.org/MAVERICLab/vcontact2/src/master/
```

```
#to prepare the gene-to-genome files which require protein files, used
prodigal
prodigal -i contigs.fasta -a contigs.aa.fasta -p meta
#default settings in vConTACT2 were used
#networks were produced in Cytoscape with the output of vConTACT2
#repeat for each rock type

#CenoteTaker2
#link: https://github.com/mtisza1/Cenote-Taker2
#repeat for each rock type

#Clinker
#link: https://github.com/gamcil/clinker
#default settings were used
#repeat for each rock type

#PHACTS
#link: https://github.com/deprekate/PHACTS
#default settings were used
#repeat for each rock type

#host prediction
#link: ARAGORN:
https://github.com/TheSEED/aragorn/blob/master/aragorn1.2.36.c
#link: CRISPR CasTyper: https://github.com/Russel88/CRISPRCasTyper
#default settings were used for ARAGORN and CRISPRCasTyper
#repeat for each rock type

#VIBRANT
#link: https://github.com/AnantharamanLab/VIBRANT
#default settings were used
#repeat for each rock type

#DRAMv
#link: https://github.com/WrightonLabCSU/DRAM/wiki/3b.-Running-DRAM-v
#default settings were used
#repeat for each rock type

#CMSeq2
#link: https://github.com/SegataLab/cmseq/blob/master/README.md
#to QC the reads, used fastp pipeline
fastp -i READS_1.fastq -I READS_2.fastq -o READS_1.trim.fastq -O
READS_2.trim.fastq -w 8
#to align reads to the fasta contigs, used bowtie2 to generate .sam files.
bowtie2 -q -p8 -x bowtie_dbs/my_virus_contigs -1 reads_1.trim.fastq -2
reads_2.trim.fastq -S reads_align_to_my_virus_contigs.sam
#to convert to .bam, sort the .bam, index the .bam, used samtools
samtools view -bS –threads 8 READS_align_to_my_virus_contigs.sam >
reads_align_to_my_virus_contigs.bam
samtools sort –threads 8 reads_align_to_my_virus_contigs.bam -o
reads_align_to_my_virus_contigs.sorted.bam
samtools index reads_align_to_my_virus_contigs.sorted.bam
#cmseq2 input
breadth_depth.py reads_align_to_my_virus_contigs.sorted.bam >
reads_align_to_my_virus_contigs.sorted.cmseq1.tsv
#repeat for each rock type
```
